# Crystal structure of di­chlorido­{*N* ^1^-phenyl-*N* ^4^-[(quinolin-2-yl-κ*N*)methylidene]benzene-1,4-diamine-κ*N* ^4^}mercury(II)

**DOI:** 10.1107/S2056989015001620

**Published:** 2015-01-31

**Authors:** Md. Serajul Haque Faizi, Elena V. Prisyazhnaya, Turganbay S. Iskenderov

**Affiliations:** a Department of Chemistry, Indian Institute of Technology Kanpur, Kanpur, UP 208 016, India; b Department of Chemistry, Kyiv National University of Construction and Architecture, Povitroflotsky Avenue 31, 03680 Kiev, Ukraine; c National Taras Shevchenko University, Department of Chemistry, Volodymyrska str. 64, 01601 Kyiv, Ukraine

**Keywords:** crystal structure, Schiff base, mercury(II) complex, N—H⋯Cl and C—H⋯Cl hydrogen bonding, π–π stacking inter­actions

## Abstract

In the mononuclear title complex, [HgCl_2_(C_22_H_17_N_3_)], synthesized from the quinoline-derived Schiff base *N* ^1^-phenyl-*N* ^4^-[(quinolin-2-yl)methyl­idene]benzene-1,4-di­amine (PQMBD) and HgCl_2_, the coordination sphere around the Hg^2+^ atom is distorted tetra­hedral, comprising two Cl atoms [Hg—Cl = 2.3487 (14) and 2.4490 (15) Å] and two N atom donors from the PQMBD ligand, *viz*. the quinolyl and the imine N atom [Hg—N = 2.270 (4) and 2.346 (4) Å, respectively]. The dihedral angle between the two benzene rings attached to the amino group is 43.7 (3)°. In the crystal, N—H⋯Cl and C—H⋯Cl hydrogen bonds, as well as π–π stacking inter­actions between one phenyl ring and the pyridine ring of the quinoline moiety of an adjacent mol­ecule [centroid-to-centroid separation = 3.617 (4) Å] are observed, resulting in a three-dimensional network.

## Related literature   

For the haza­rds of mercury in organisms, see: Mandal *et al.* (2012 ▸). For reports of quinolyl derivatives of Schiff bases, see: Motswainyana *et al.* (2013 ▸); Das *et al.* (2013 ▸); Song *et al.* (2011 ▸); Jursic *et al.* (2002 ▸). For background to related Schiff base–metal complexes, see: Faizi & Hussain (2014 ▸); Faizi *et al.* (2014 ▸); Moroz *et al.* (2012 ▸). For related Hg-containing structures, see: Marjani *et al.* (2009 ▸); Faizi & Sen (2014 ▸), and for related Schiff base complexes, see: Penkova *et al.* (2009 ▸, 2010 ▸); Strotmeyer *et al.* (2003 ▸); Petrusenko *et al.* (1997 ▸). The amino group of the title compound is separated from the chelating unit which makes this complex a possible precursor for further functionalization, eventually yielding binuclear compounds as reported by Fritsky *et al.* (1998 ▸, 2006 ▸) and Kanderal *et al.* (2005 ▸).
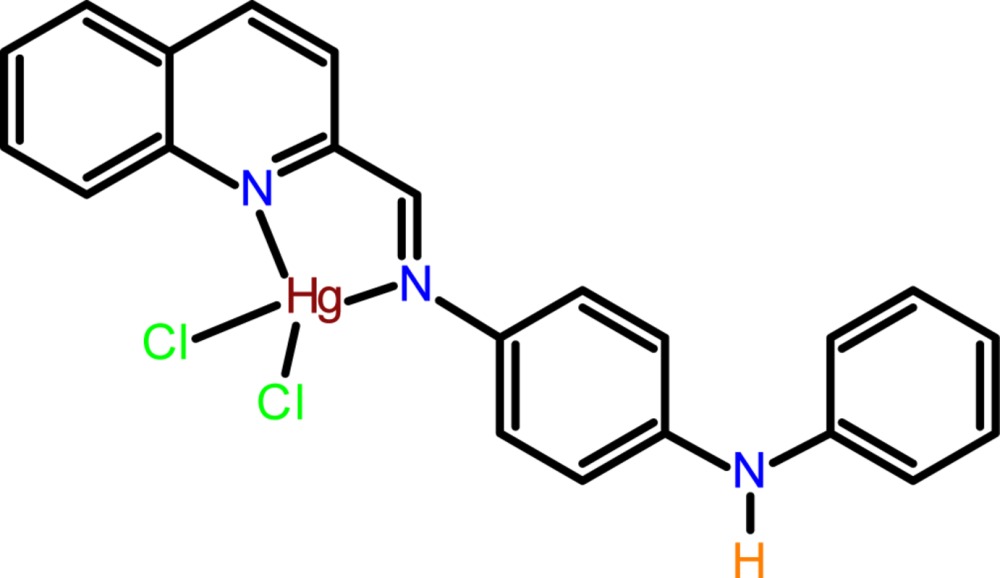


## Experimental   

### Crystal data  

[HgCl_2_(C_22_H_17_N_3_)]
*M* *_r_* = 594.88
Monoclinic, 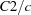

*a* = 29.265 (5) Å
*b* = 7.5671 (13) Å
*c* = 18.811 (3) Å
β = 99.271 (7)°
*V* = 4111.4 (12) Å^3^
*Z* = 8
Mo *K*α radiation
μ = 7.76 mm^−1^
*T* = 100 K
0.18 × 0.15 × 0.10 mm

### Data collection  

Bruker SMART APEX CCD diffractometer
Absorption correction: multi-scan (*SADABS*; Bruker, 2003 ▸) *T*_min_ = 0.336, *T*_max_ = 0.511
22545 measured reflections
5133 independent reflections
3182 reflections with *I* > 2σ(*I*)
*R*_int_ = 0.059

### Refinement  

*R*[*F* ^2^ > 2σ(*F* ^2^)] = 0.038
*wR*(*F* ^2^) = 0.087
*S* = 1.00
5133 reflections
253 parameters
H-atom parameters constrained
Δρ_max_ = 0.99 e Å^−3^
Δρ_min_ = −0.56 e Å^−3^

### 

Data collection: *SMART* (Bruker, 2003 ▸); cell refinement: *SAINT* (Bruker, 2003 ▸); data reduction: *SAINT*; program(s) used to solve structure: *SIR97* (Altomare *et al.*, 1999 ▸); program(s) used to refine structure: *SHELXL97* (Sheldrick, 2015 ▸); molecular graphics: *DIAMOND* (Brandenburg & Putz, 2006 ▸) and *Mercury* (Macrae *et al.*, 2008 ▸); software used to prepare material for publication: *SHELXL97* and *PLATON* (Spek, 2009 ▸).

## Supplementary Material

Crystal structure: contains datablock(s) global, I. DOI: 10.1107/S2056989015001620/wm5117sup1.cif

Structure factors: contains datablock(s) I. DOI: 10.1107/S2056989015001620/wm5117Isup2.hkl

**Fig. 1.**
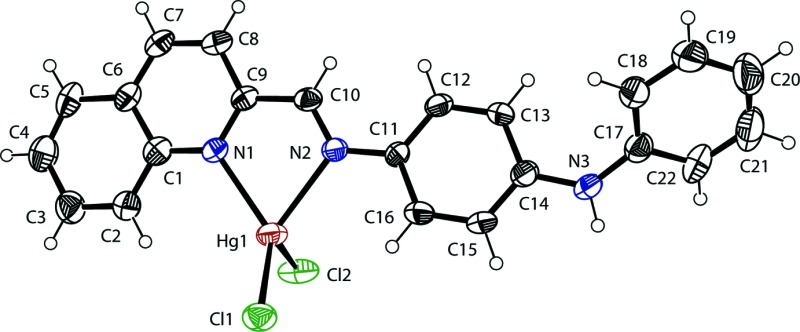
The molecular structure and the atom-numbering scheme of the title complex, with non-H atoms drawn as displacement ellipsoids at the 40% probability level.

. DOI: 10.1107/S2056989015001620/wm5117fig1.tif
The mol­ecular structure and the atom-numbering scheme of the title complex, with non-H atoms drawn as displacement ellipsoids at the 40% probability level.

**Fig. 2.**
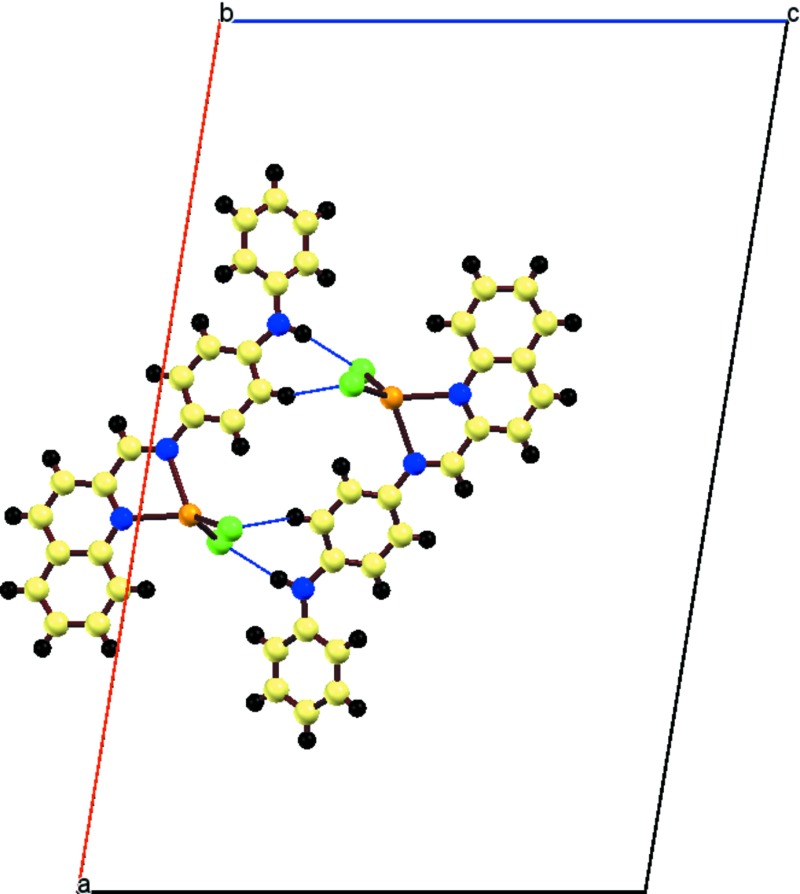
N—H···Cl hydrogen bonds between adjacent molecules as viewed along [010].

. DOI: 10.1107/S2056989015001620/wm5117fig2.tif
N—H⋯Cl hydrogen bonds between adjacent mol­ecules as viewed along [010].

**Fig. 3.**
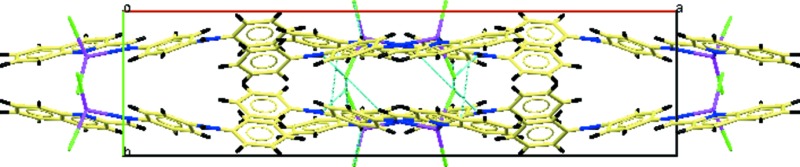
The packing of molecules in the title compound, showing intermolecular interactions as dashed lines.

. DOI: 10.1107/S2056989015001620/wm5117fig3.tif
The packing of mol­ecules in the title compound, showing inter­molecular inter­actions as dashed lines.

CCDC reference: 1045457

Additional supporting information:  crystallographic information; 3D view; checkCIF report

## Figures and Tables

**Table 1:** Hydrogen-bond geometry (, )

*D*H*A*	*D*H	H*A*	*D* *A*	*D*H*A*
N3H3*A*Cl2^i^	0.86	2.58	3.363(4)	151
C10H10Cl2^ii^	0.93	2.81	3.679(7)	157
C20H20Cl1^iii^	0.93	2.80	3.692(11)	160

Symmetry codes: (i) 
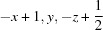
; (ii) 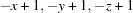; (iii) 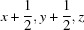.

## supplementary crystallographic information

### S1. Experimental

The iminoquinolyl ligand *N*^1^-phenyl-*N*^4^-[(quinolin-2-yl)methylidene]benzene-1,4-diamine (PQMBD) was prepared by reacting 2-quinolinecarboxaldehyde (0.085 g, 0.54 mmol) with one equivalent of *N*-phenyl-*p*-phenylenediamine (0.100 g, 0.54 mmol) and was obtained in 88% yield (0.15 g). The obtained compound was characterized by FT–IR, NMR and ESI-mass spectroscopy: IR (KBr, ν / cm^-1^): 3417, 3052 (C-H arom), 1620 (C═N), 1515, 1313, 843, 756. ^1^H NMR (400 MHz, CDCl_3_, δ /p.p.m.): 8.85 (1H, S), 8.37 (1H, d), 8.23 (1H, d), 8.16 (1H, d), 7.86 (1H, d), 7.75 (1H, t), 7.58 (1H, t), 7.40 (2H, d), 7.30 (1H, t), 7.13 (5H, m), 6.51 (1H, t). ESI-MS m/z: 324 (M+1).

PQMBD (0.10 g, 0.31 mmol), mercury(II) chloride (0.08 g, 0.31 mmol) and ethanol (5 ml) were stirred vigorously for 1 h, after which the precipitate was filtered off and redissolved in dimethylformamide. Crystals of the title complex suitable for X-ray analysis was obtained within 3 days by slow evaporation of the DMF solvent.

### S2. Refinement

The N-bound H-atom was located in a difference Fourier maps, and the positions restrained to N—H = 0.86 Å and *U*_iso_(H) = 1.2*U*_eq_(N). All other H-atoms were positioned geometrically and refined using a riding model with C—H = 0.93 Å and *U*_iso_(H) = 1.2*U*_eq_(C).

### Crystal data

**Table:**

[HgCl_2_(C_22_H_17_N_3_)]	*F*(000) = 2272
*M**_r_* = 594.88	*D*_x_ = 1.922 Mg m^−^^3^
Monoclinic, *C*2/*c*	Mo *K*α radiation, λ = 0.71073 Å
Hall symbol: -C 2yc	Cell parameters from 7479 reflections
*a* = 29.265 (5) Å	θ = 2.8–24.6°
*b* = 7.5671 (13) Å	µ = 7.76 mm^−^^1^
*c* = 18.811 (3) Å	*T* = 100 K
β = 99.271 (7)°	Block, colourless
*V* = 4111.4 (12) Å^3^	0.18 × 0.15 × 0.10 mm
*Z* = 8	

### Data collection

**Table:**

Bruker SMART APEX CCD diffractometer	5133 independent reflections
Radiation source: fine-focus sealed tube	3182 reflections with *I* > 2σ(*I*)
Graphite monochromator	*R*_int_ = 0.059
ω scans	θ_max_ = 28.4°, θ_min_ = 2.8°
Absorption correction: multi-scan (*SADABS*; Bruker, 2003)	*h* = −38→38
*T*_min_ = 0.336, *T*_max_ = 0.511	*k* = −10→9
22545 measured reflections	*l* = −25→25

### Refinement

**Table:**

Refinement on *F*^2^	Primary atom site location: structure-invariant direct methods
Least-squares matrix: full	Secondary atom site location: difference Fourier map
*R*[*F*^2^ > 2σ(*F*^2^)] = 0.038	Hydrogen site location: inferred from neighbouring sites
*wR*(*F*^2^) = 0.087	H-atom parameters constrained
*S* = 1.00	*w* = 1/[σ^2^(*F*_o_^2^) + (0.0374*P*)^2^ + 1.5818*P*] where *P* = (*F*_o_^2^ + 2*F*_c_^2^)/3
5133 reflections	(Δ/σ)_max_ = 0.001
253 parameters	Δρ_max_ = 0.99 e Å^−^^3^
0 restraints	Δρ_min_ = −0.56 e Å^−^^3^

### Special details

**Table:**

Geometry. All e.s.d.'s (except the e.s.d. in the dihedral angle between two l.s. planes) are estimated using the full covariance matrix. The cell e.s.d.'s are taken into account individually in the estimation of e.s.d.'s in distances, angles and torsion angles; correlations between e.s.d.'s in cell parameters are only used when they are defined by crystal symmetry. An approximate (isotropic) treatment of cell e.s.d.'s is used for estimating e.s.d.'s involving l.s. planes.
Refinement. Refinement of *F*^2^ against ALL reflections. The weighted *R*-factor *wR* and goodness of fit *S* are based on *F*^2^, conventional *R*-factors *R* are based on *F*, with *F* set to zero for negative *F*^2^. The threshold expression of *F*^2^ > σ(*F*^2^) is used only for calculating *R*-factors(gt) *etc*. and is not relevant to the choice of reflections for refinement. *R*-factors based on *F*^2^ are statistically about twice as large as those based on *F*, and *R*- factors based on ALL data will be even larger.

### Fractional atomic coordinates and isotropic or equivalent isotropic displacement parameters (Å^2^)

**Table:**

	*x*	*y*	*z*	*U*_iso_*/*U*_eq_	
N1	0.42861 (15)	0.2191 (5)	0.5298 (2)	0.0396 (10)	
N2	0.50810 (14)	0.2420 (6)	0.4703 (2)	0.0389 (10)	
N3	0.65162 (17)	0.3036 (7)	0.3095 (2)	0.0603 (14)	
H3A	0.6410	0.3037	0.2641	0.072*	
C11	0.54666 (17)	0.2526 (7)	0.4348 (2)	0.0373 (12)	
C10	0.50915 (18)	0.2791 (7)	0.5369 (3)	0.0422 (13)	
H10	0.5369	0.3144	0.5644	0.051*	
C17	0.6984 (2)	0.3274 (7)	0.3265 (3)	0.0507 (14)	
C12	0.59004 (18)	0.3146 (7)	0.4656 (2)	0.0431 (13)	
H12	0.5952	0.3467	0.5140	0.052*	
C1	0.3891 (2)	0.2106 (7)	0.5581 (3)	0.0475 (14)	
C16	0.5400 (2)	0.2029 (7)	0.3633 (3)	0.0509 (15)	
H16	0.5115	0.1588	0.3418	0.061*	
C9	0.46781 (18)	0.2673 (7)	0.5702 (3)	0.0392 (12)	
C15	0.5752 (2)	0.2181 (8)	0.3236 (3)	0.0572 (16)	
H15	0.5697	0.1861	0.2753	0.069*	
C13	0.62510 (18)	0.3293 (7)	0.4263 (3)	0.0447 (13)	
H13	0.6537	0.3729	0.4481	0.054*	
C6	0.3892 (2)	0.2468 (8)	0.6324 (3)	0.0479 (14)	
C7	0.4309 (2)	0.2985 (7)	0.6737 (3)	0.0543 (15)	
H7	0.4319	0.3271	0.7220	0.065*	
C8	0.4699 (2)	0.3076 (8)	0.6443 (3)	0.0559 (16)	
H8	0.4978	0.3400	0.6722	0.067*	
C14	0.61872 (19)	0.2795 (7)	0.3533 (3)	0.0469 (14)	
Hg1	0.432753 (8)	0.18401 (3)	0.411137 (10)	0.05198 (10)	
Cl2	0.40186 (6)	0.4552 (2)	0.35050 (7)	0.0702 (5)	
Cl1	0.41693 (5)	−0.0647 (2)	0.33652 (7)	0.0593 (4)	
C2	0.3473 (2)	0.1623 (7)	0.5149 (3)	0.0553 (15)	
H2	0.3471	0.1343	0.4667	0.066*	
C4	0.3076 (2)	0.1986 (8)	0.6148 (4)	0.0699 (19)	
H4	0.2798	0.1988	0.6327	0.084*	
C3	0.3070 (2)	0.1557 (8)	0.5420 (3)	0.0656 (18)	
H3	0.2795	0.1233	0.5129	0.079*	
C5	0.3471 (2)	0.2397 (9)	0.6601 (3)	0.0624 (17)	
H5	0.3465	0.2628	0.7085	0.075*	
C18	0.7240 (2)	0.2570 (9)	0.3877 (3)	0.0632 (17)	
H18	0.7096	0.1968	0.4210	0.076*	
C19	0.7715 (3)	0.2770 (13)	0.3989 (4)	0.098 (3)	
H19	0.7890	0.2272	0.4396	0.118*	
C22	0.7218 (3)	0.4179 (9)	0.2794 (3)	0.0700 (19)	
H22	0.7050	0.4696	0.2384	0.084*	
C21	0.7680 (3)	0.4324 (11)	0.2915 (5)	0.091 (3)	
H21	0.7826	0.4886	0.2573	0.110*	
C20	0.7937 (3)	0.3686 (15)	0.3512 (5)	0.115 (4)	
H20	0.8256	0.3856	0.3602	0.138*	

### Atomic displacement parameters (Å^2^)

**Table:**

	*U*^11^	*U*^22^	*U*^33^	*U*^12^	*U*^13^	*U*^23^
N1	0.046 (3)	0.040 (3)	0.033 (2)	0.002 (2)	0.0060 (19)	0.0038 (18)
N2	0.043 (3)	0.040 (2)	0.033 (2)	−0.003 (2)	0.0032 (18)	−0.0005 (18)
N3	0.050 (3)	0.101 (4)	0.030 (2)	0.000 (3)	0.007 (2)	0.001 (2)
C11	0.039 (3)	0.041 (3)	0.034 (3)	0.001 (2)	0.011 (2)	−0.006 (2)
C10	0.045 (3)	0.044 (3)	0.034 (3)	0.003 (2)	−0.002 (2)	0.007 (2)
C17	0.053 (4)	0.056 (4)	0.046 (3)	0.003 (3)	0.015 (3)	−0.004 (3)
C12	0.050 (3)	0.050 (3)	0.029 (2)	−0.002 (3)	0.003 (2)	−0.002 (2)
C1	0.055 (4)	0.040 (3)	0.047 (3)	0.009 (3)	0.008 (3)	0.008 (2)
C16	0.048 (3)	0.064 (4)	0.039 (3)	−0.007 (3)	0.000 (2)	−0.014 (3)
C9	0.048 (3)	0.038 (3)	0.033 (3)	0.007 (2)	0.007 (2)	0.000 (2)
C15	0.055 (4)	0.085 (5)	0.030 (3)	−0.009 (3)	0.002 (3)	−0.013 (3)
C13	0.037 (3)	0.058 (4)	0.037 (3)	−0.005 (3)	0.000 (2)	−0.007 (3)
C6	0.057 (4)	0.048 (3)	0.042 (3)	0.006 (3)	0.015 (3)	0.009 (3)
C7	0.070 (4)	0.061 (4)	0.034 (3)	0.002 (3)	0.015 (3)	−0.003 (3)
C8	0.060 (4)	0.071 (4)	0.034 (3)	−0.001 (3)	0.000 (3)	0.003 (3)
C14	0.046 (3)	0.058 (4)	0.037 (3)	0.005 (3)	0.007 (2)	0.000 (3)
Hg1	0.05736 (16)	0.05833 (16)	0.03782 (13)	−0.00317 (12)	0.00037 (9)	−0.00853 (11)
Cl2	0.1027 (13)	0.0504 (9)	0.0476 (8)	0.0032 (9)	−0.0178 (8)	−0.0030 (7)
Cl1	0.0732 (10)	0.0508 (9)	0.0524 (8)	−0.0083 (8)	0.0053 (7)	−0.0122 (7)
C2	0.052 (4)	0.064 (4)	0.052 (3)	−0.002 (3)	0.012 (3)	0.000 (3)
C4	0.066 (4)	0.078 (5)	0.073 (5)	0.007 (4)	0.032 (4)	−0.001 (4)
C3	0.049 (4)	0.078 (5)	0.070 (4)	0.003 (3)	0.011 (3)	0.007 (4)
C5	0.069 (5)	0.068 (4)	0.058 (4)	0.009 (4)	0.034 (4)	0.010 (3)
C18	0.051 (4)	0.087 (5)	0.052 (4)	0.003 (4)	0.010 (3)	0.009 (3)
C19	0.057 (5)	0.174 (9)	0.062 (4)	0.006 (5)	0.006 (4)	−0.022 (5)
C22	0.082 (5)	0.076 (5)	0.061 (4)	0.009 (4)	0.040 (4)	0.008 (4)
C21	0.094 (6)	0.097 (6)	0.098 (6)	−0.031 (5)	0.061 (5)	−0.025 (5)
C20	0.069 (6)	0.179 (10)	0.107 (7)	−0.036 (6)	0.045 (5)	−0.069 (7)

### Geometric parameters (Å, º)

**Table:**

N1—C9	1.322 (6)	C13—H13	0.9300
N1—C1	1.349 (7)	C6—C7	1.394 (8)
N1—Hg1	2.270 (4)	C6—C5	1.413 (8)
N2—C10	1.279 (6)	C7—C8	1.347 (8)
N2—C11	1.402 (6)	C7—H7	0.9300
N2—Hg1	2.346 (4)	C8—H8	0.9300
N3—C17	1.367 (7)	Hg1—Cl1	2.3487 (14)
N3—C14	1.377 (7)	Hg1—Cl2	2.4490 (15)
N3—H3A	0.8600	C2—C3	1.359 (8)
C11—C16	1.380 (6)	C2—H2	0.9300
C11—C12	1.389 (7)	C4—C5	1.358 (9)
C10—C9	1.453 (7)	C4—C3	1.403 (8)
C10—H10	0.9300	C4—H4	0.9300
C17—C22	1.385 (8)	C3—H3	0.9300
C17—C18	1.375 (8)	C5—H5	0.9300
C12—C13	1.362 (7)	C18—C19	1.381 (9)
C12—H12	0.9300	C18—H18	0.9300
C1—C2	1.404 (8)	C19—C20	1.375 (12)
C1—C6	1.425 (7)	C19—H19	0.9300
C16—C15	1.370 (8)	C22—C21	1.340 (9)
C16—H16	0.9300	C22—H22	0.9300
C9—C8	1.418 (7)	C21—C20	1.338 (11)
C15—C14	1.385 (7)	C21—H21	0.9300
C15—H15	0.9300	C20—H20	0.9300
C13—C14	1.409 (7)		
			
C9—N1—C1	120.4 (4)	C6—C7—H7	119.7
C9—N1—Hg1	114.8 (3)	C7—C8—C9	119.1 (5)
C1—N1—Hg1	124.5 (4)	C7—C8—H8	120.4
C10—N2—C11	124.0 (4)	C9—C8—H8	120.4
C10—N2—Hg1	112.2 (3)	C15—C14—N3	119.3 (5)
C11—N2—Hg1	123.5 (3)	C15—C14—C13	116.8 (5)
C17—N3—C14	130.3 (5)	N3—C14—C13	123.6 (5)
C17—N3—H3A	114.8	N1—Hg1—N2	72.96 (15)
C14—N3—H3A	114.8	N1—Hg1—Cl1	130.14 (11)
C16—C11—C12	118.3 (5)	N2—Hg1—Cl1	120.86 (11)
C16—C11—N2	116.8 (5)	N1—Hg1—Cl2	106.60 (11)
C12—C11—N2	124.9 (4)	N2—Hg1—Cl2	108.21 (11)
N2—C10—C9	121.3 (5)	Cl1—Hg1—Cl2	111.75 (5)
N2—C10—H10	119.4	C3—C2—C1	121.4 (6)
C9—C10—H10	119.4	C3—C2—H2	119.3
N3—C17—C22	119.6 (5)	C1—C2—H2	119.3
N3—C17—C18	122.4 (5)	C5—C4—C3	122.7 (6)
C22—C17—C18	118.0 (6)	C5—C4—H4	118.7
C13—C12—C11	121.2 (4)	C3—C4—H4	118.7
C13—C12—H12	119.4	C2—C3—C4	118.9 (6)
C11—C12—H12	119.4	C2—C3—H3	120.6
N1—C1—C2	120.5 (5)	C4—C3—H3	120.6
N1—C1—C6	120.8 (5)	C4—C5—C6	118.8 (6)
C2—C1—C6	118.7 (5)	C4—C5—H5	120.6
C15—C16—C11	120.5 (5)	C6—C5—H5	120.6
C15—C16—H16	119.7	C19—C18—C17	119.0 (6)
C11—C16—H16	119.7	C19—C18—H18	120.5
N1—C9—C8	121.4 (5)	C17—C18—H18	120.5
N1—C9—C10	118.3 (4)	C18—C19—C20	121.7 (8)
C8—C9—C10	120.3 (5)	C18—C19—H19	119.2
C16—C15—C14	122.1 (5)	C20—C19—H19	119.2
C16—C15—H15	118.9	C21—C22—C17	121.4 (7)
C14—C15—H15	118.9	C21—C22—H22	119.3
C12—C13—C14	121.0 (5)	C17—C22—H22	119.3
C12—C13—H13	119.5	C22—C21—C20	121.9 (7)
C14—C13—H13	119.5	C22—C21—H21	119.0
C7—C6—C5	122.9 (5)	C20—C21—H21	119.0
C7—C6—C1	117.6 (5)	C21—C20—C19	118.0 (8)
C5—C6—C1	119.4 (6)	C21—C20—H20	121.0
C8—C7—C6	120.6 (5)	C19—C20—H20	121.0
C8—C7—H7	119.7		
			
C10—N2—C11—C16	178.2 (5)	C16—C15—C14—N3	−175.4 (6)
Hg1—N2—C11—C16	−8.8 (7)	C16—C15—C14—C13	−1.1 (9)
C10—N2—C11—C12	−4.2 (8)	C17—N3—C14—C15	−165.2 (6)
Hg1—N2—C11—C12	168.9 (4)	C17—N3—C14—C13	20.9 (10)
C11—N2—C10—C9	179.3 (5)	C12—C13—C14—C15	0.9 (8)
Hg1—N2—C10—C9	5.6 (6)	C12—C13—C14—N3	174.9 (5)
C14—N3—C17—C22	−153.7 (6)	C9—N1—Hg1—N2	5.3 (3)
C14—N3—C17—C18	29.6 (10)	C1—N1—Hg1—N2	178.8 (4)
C16—C11—C12—C13	1.1 (8)	C9—N1—Hg1—Cl1	121.6 (3)
N2—C11—C12—C13	−176.5 (5)	C1—N1—Hg1—Cl1	−64.9 (4)
C9—N1—C1—C2	178.9 (5)	C9—N1—Hg1—Cl2	−99.2 (3)
Hg1—N1—C1—C2	5.8 (7)	C1—N1—Hg1—Cl2	74.3 (4)
C9—N1—C1—C6	−2.3 (8)	C10—N2—Hg1—N1	−5.6 (3)
Hg1—N1—C1—C6	−175.5 (4)	C11—N2—Hg1—N1	−179.4 (4)
C12—C11—C16—C15	−1.3 (8)	C10—N2—Hg1—Cl1	−132.7 (3)
N2—C11—C16—C15	176.6 (5)	C11—N2—Hg1—Cl1	53.5 (4)
C1—N1—C9—C8	1.3 (7)	C10—N2—Hg1—Cl2	96.7 (4)
Hg1—N1—C9—C8	175.1 (4)	C11—N2—Hg1—Cl2	−77.1 (4)
C1—N1—C9—C10	−178.3 (5)	N1—C1—C2—C3	−178.9 (5)
Hg1—N1—C9—C10	−4.6 (6)	C6—C1—C2—C3	2.3 (8)
N2—C10—C9—N1	−0.9 (8)	C1—C2—C3—C4	0.1 (9)
N2—C10—C9—C8	179.5 (5)	C5—C4—C3—C2	−2.7 (10)
C11—C16—C15—C14	1.3 (10)	C3—C4—C5—C6	2.7 (10)
C11—C12—C13—C14	−1.0 (8)	C7—C6—C5—C4	175.9 (6)
N1—C1—C6—C7	2.8 (8)	C1—C6—C5—C4	−0.1 (9)
C2—C1—C6—C7	−178.5 (5)	N3—C17—C18—C19	175.8 (6)
N1—C1—C6—C5	178.9 (5)	C22—C17—C18—C19	−0.9 (10)
C2—C1—C6—C5	−2.3 (8)	C17—C18—C19—C20	1.4 (12)
C5—C6—C7—C8	−178.2 (6)	N3—C17—C22—C21	−174.9 (6)
C1—C6—C7—C8	−2.2 (9)	C18—C17—C22—C21	1.9 (10)
C6—C7—C8—C9	1.3 (9)	C17—C22—C21—C20	−3.5 (13)
N1—C9—C8—C7	−0.8 (8)	C22—C21—C20—C19	3.8 (14)
C10—C9—C8—C7	178.9 (5)	C18—C19—C20—C21	−2.8 (14)

### Hydrogen-bond geometry (Å, º)

**Table:**

*D*—H···*A*	*D*—H	H···*A*	*D*···*A*	*D*—H···*A*
N3—H3*A*···Cl2^i^	0.86	2.58	3.363 (4)	151
C10—H10···Cl2^ii^	0.93	2.81	3.679 (7)	157
C20—H20···Cl1^iii^	0.93	2.80	3.692 (11)	160

Symmetry codes: (i) −*x*+1, *y*, −*z*+1/2; (ii) −*x*+1, −*y*+1, −*z*+1; (iii) *x*+1/2, *y*+1/2, *z*.
