# Supplementary figures and images for: Crystal structure of di­chlorido­{N 1-phenyl-N 4-[(quinolin-2-yl-κN)methylidene]benzene-1,4-diamine-κN 4}mercury(II)
Source: Acta Crystallogr E Crystallogr Commun. 2015 Jan 31;71(Pt 2):m46–7. doi: 10.1107/S2056989015001620 (PMC4384595; doi:10.1107/S2056989015001620)

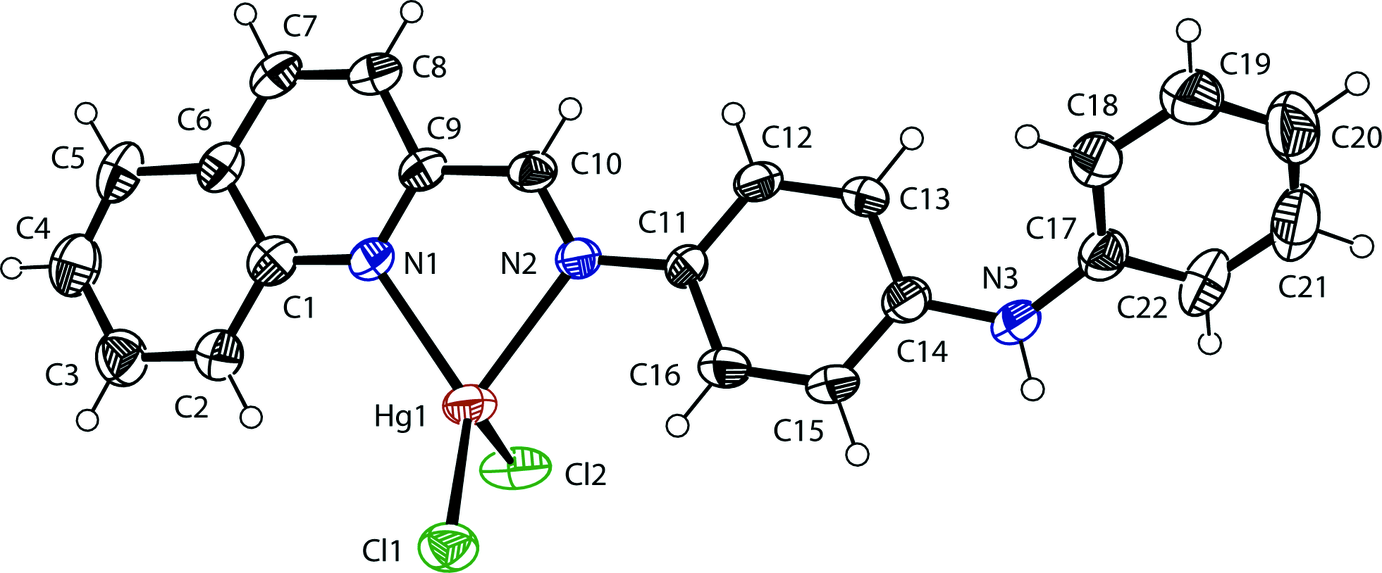

Supplement: Supplementary file 3 [file e-71-00m46-fig1.tif]

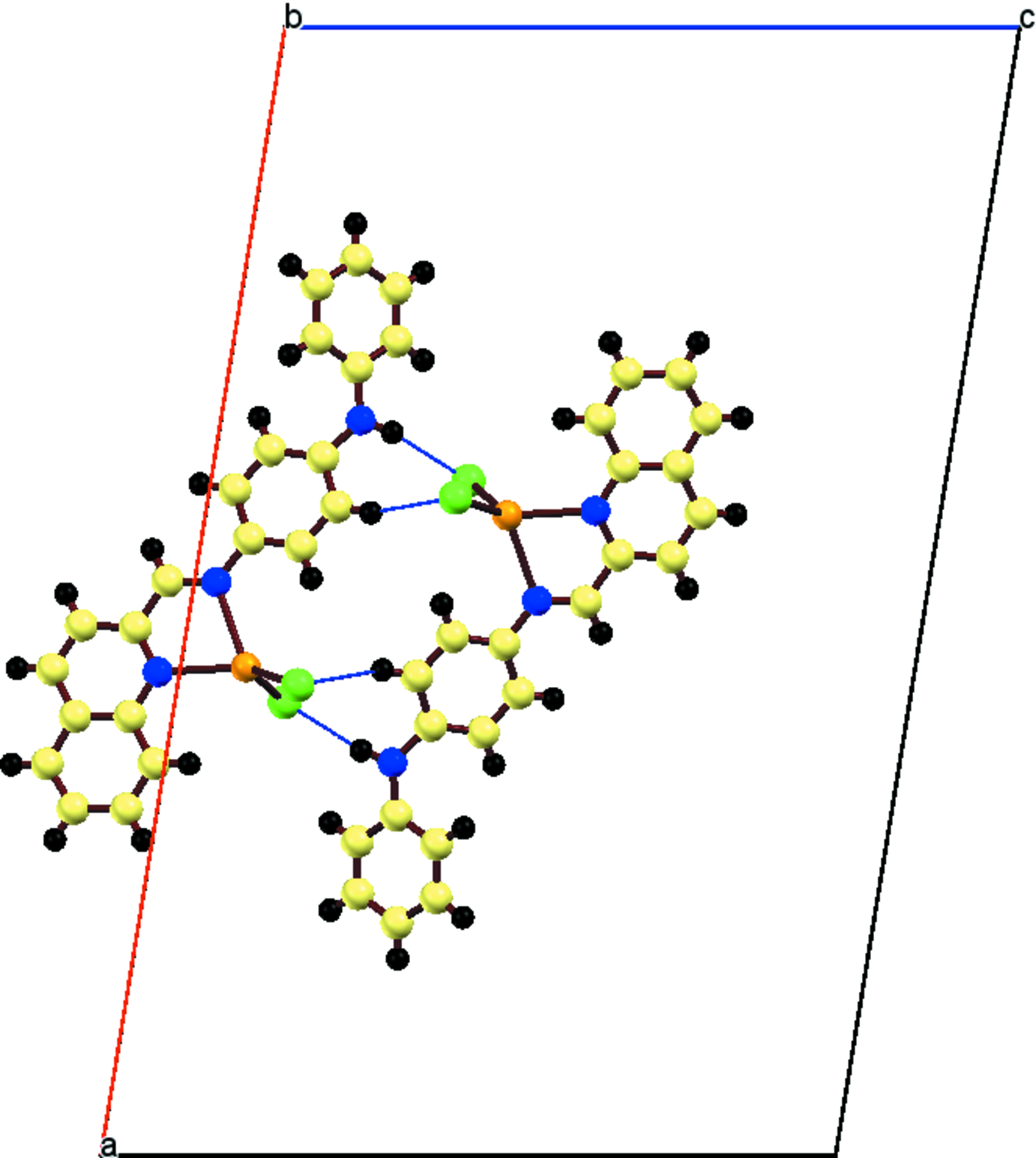

Supplement: Supplementary file 4 [file e-71-00m46-fig2.tif]

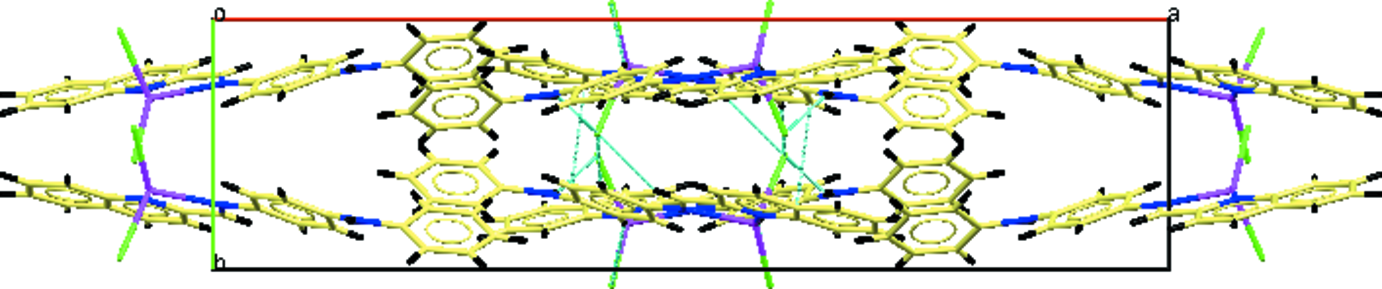

Supplement: Supplementary file 5 [file e-71-00m46-fig3.tif]
